# Modifiable risk factors and their joint effect on Schizophrenia: A perspective study

**DOI:** 10.1192/j.eurpsy.2025.1977

**Published:** 2025-08-26

**Authors:** S. Chen, Q. Dong, E. Fernandez-Egea, X. Li, A. Dayimu, D. Liu, H. Wang, F. Jiang, L. Xu

**Affiliations:** 1 London School of Hygiene & Tropical Medicine, London, United Kingdom; 2 Shandong University, Shandong, China; 3 University of Cambridge, Cambridge, United Kingdom

## Abstract

**Introduction:**

Schizophrenia is a severe psychiatric disorder affecting 50% of patients intermittently and 20% chronically, with high unemployment rates (80-90%) and reduced life expectancy. Although genetic and neurodevelopmental factors are established non-modifiable risk factors, knowledge gaps persist regarding prevention strategies, particularly the combined impact of modifiable risk factors.

**Objectives:**

The aim of this study is to identify the modifiable risk factors and to estimate their joint effect on Schizophrenia.

**Methods:**

We conducted an exposure-wide association study (EWAS) using the UK Biobank cohort to systematically evaluate 206 potentially modifiable factors associated with schizophrenia risk. The study population comprised individuals without schizophrenia at baseline, with diagnoses determined using ICD-10 criteria. We employed Cox proportional hazard regression models with Bonferroni correction (significance threshold: P<1.91×10^-4^) to identify significant factors. The identified factors were categorized into six domains: lifestyle, local environment, medical history, physical measures, psychosocial factors, and socioeconomic status (SES). Domain-specific, weighed, and standardized scores were calculated based on coefficients from Cox models, adjusting for covariates. Scores were stratified into tertiles (favorable, intermediate, unfavorable) for risk assessment. Population attributable fractions (PAFs) were calculated to quantify prevention potential.

**Results:**

The study cohort included 498,351 participants (54.45% female; mean age: 56.55 years) followed for a mean duration of 14.37 years, during which 1,345 participants developed schizophrenia. We identified 86 significant modifiable factors, with disability (HR 6.23, 95% CI 5.48-7.07), depression (HR 5.06, 95% CI 4.93-5.20), and anxiety disorders (HR 3.69, 95% CI 3.12-4.36) showing the strongest associations. Our analyses suggested that transitioning unfavorable profiles to intermediate and favorable status (Estimation 1) could prevent 59.6% of schizophrenia cases, while shifting both intermediate and unfavorable profiles to favorable (Estimation 2) could prevent 90.4% of cases. In Estimation 2, the preventive potential was highest for SES (18.0%), followed by medical history (17.5%), lifestyle factors (17.0%), psychosocial factors (14.3%), physical measures (12.8%), and local environment (10.8%).

**Image:**

**Figure:**
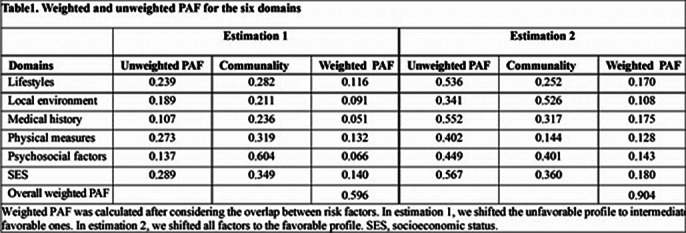

**Conclusions:**

This analysis identifies multiple modifiable risk factors for schizophrenia, demonstrating substantial prevention potential through multi-domain interventions. Socioeconomic, medical, and lifestyle factors emerge as key targets for prevention strategies. The consistency of associations across genetic risk strata suggests interventions could be beneficial regardless of genetic predisposition, informing targeted prevention strategies and public health policies.

**Disclosure of Interest:**

None Declared

